# Mortality Trends in the United States of America Among Adults With Thyroid Disorders and Cardiac Arrest as a Contributing Cause: A Retrospective Observational Study

**DOI:** 10.7759/cureus.96997

**Published:** 2025-11-16

**Authors:** Gourav Gourisaria, Sonali Prasad, Andrew A James, Sanny S Samson

**Affiliations:** 1 Acute Internal Medicine, Sandwell and West Birmingham Hospitals NHS Trust, West Midlands, GBR; 2 Critical Care, Teerthanker Mahaveer University, Moradabad, IND; 3 Surgery, The University of the West Indies, Kingston, JAM; 4 Surgery, University Hospitals Birmingham NHS Foundation Trust, Birmingham, GBR

**Keywords:** cardiac arrest, cardiovascular disease, cdc wonder, mortality trends, thyroid disorders

## Abstract

Background

Thyroid disorders can influence cardiovascular function, often leading to complications such as arrhythmias and cardiac arrest. However, mortality trends in adults with thyroid disorders and cardiac arrest as a contributing cause have not been well described.

Objective

This study aimed to evaluate temporal mortality trends in adults in the USA with thyroid disorders and cardiac arrest, considering demographics and geography, from 1999 to 2020.

Methodology

A retrospective observational study was conducted using the Centers for Disease Control and Prevention Wide-ranging ONline Data for Epidemiologic Research (CDC WONDER) multiple cause of death database. Deaths with thyroid disorders (International Classification of Diseases, 10th Revision or ICD-10: E00-E07) as the underlying cause and cardiac arrest (ICD-10: I46) as a contributing cause were analyzed among individuals aged ≥25 years. Mortality rates were age-adjusted to the 2000 U.S. standard population, and temporal trends were expressed as annual percentage change (APC).

Results

Between 1999 and 2020, 7,829 deaths were identified, corresponding to a crude rate of 1.7 per 1,000,000. Females accounted for 74.9% of deaths, and White individuals represented 84.1%. Most deaths occurred in metropolitan areas (82%) and medical facilities (42%). Age-adjusted mortality rates declined from 1999-2009 (APC: -5.0%, p<0.05) and gradually increased from 2009-2020 (APC: +1.2%, p<0.05).

Conclusion

Mortality associated with thyroid disorders and cardiac arrest has shown shifting trends and disparities by sex, race, and geography. Continued efforts are needed to understand contributing factors and improve prevention strategies.

## Introduction

Thyroid disorders are a noteworthy group of endocrine diseases that require clinicians to invest time in knowing how to diagnose and treat them. These can be subdivided into hyperthyroidism, with a prevalence in the US of 1.3%, and hypothyroidism, with a prevalence of 4.6% [1]. These results were derived from the National Health and Nutrition Examination Survey (NHANES) III from 1988 to 1994, with the current prevalence being relatively unknown. However, factors that increase the risk of these disorders include increasing age, obesity, alcohol, and iodine deficiency. White Americans are more affected than Black and Hispanic Americans with hypothyroidism, while the reverse is true in the case of hyperthyroidism [2].

Cardiac arrest affects many individuals in the US, approximately 600,000 individuals per year. There is a significant difference between in-hospital and out-of-hospital events, with the latter being higher, at over 340,000 per year [3]. There is also a racial disparity, with the Black American population having a higher incidence [3,4]. In addition, the Hispanic American population has a lower incidence when compared to other racial groups.

The correlation between thyroid disorders and cardiac dysfunction is well established. Excessive thyroid hormones in the blood are known to contribute to the development of varying cardiac pathologies. These include atrial fibrillation, cardiac failure, and left ventricular hypertrophy [5]. This study aims to uncover the correlation in mortality between these two conditions.

Given the established association between thyroid dysfunction and cardiovascular complications, including arrhythmias and cardiac arrest, it is essential to understand how these comorbidities influence mortality patterns at the population level. This study, therefore, aimed to examine mortality trends among adults with thyroid disorders where cardiac arrest was listed as a contributing cause of death. The specific objectives of this retrospective observational study are to: (1) evaluate temporal trends in age-adjusted mortality rates (AAMR) and annual percentage change (APC) between 1999 and 2020; (2) identify differences in mortality by sex, race, geographic region, and place of death; and (3) describe the overall demographic and geographic distribution of deaths in this population using data from the Centers for Disease Control and Prevention Wide-ranging ONline Data for Epidemiologic Research (CDC WONDER) Multiple Cause of Death (MCD) database. These data are descriptive and exploratory, intended to provide insight into the mortality disparities and inform future public health and clinical strategies.

## Materials and methods

This study was designed as a retrospective population-based study utilizing publicly available data from the CDC WONDER MCD database [6]. The MCD database compiles national mortality and population data derived from death certificates filed in all 50 U.S. states and the District of Columbia. Each death certificate includes one underlying cause of death and up to 20 contributing causes, all coded according to the International Classification of Diseases, 10th Revision (ICD-10). Because the dataset is publicly available and de-identified, no ethics committee approval was required, as per CDC policies [7].

Deaths were queried from the “Multiple Cause of Death, 1999-2020” dataset in CDC WONDER on July 25, 2025, by selecting ICD-10 codes E00-E07 (thyroid disorders) as the underlying cause of death and I46 (cardiac arrest) as a multiple/contributing cause. The query was restricted to individuals aged ≥25 years, both sexes, and all races and geographic regions. Data were extracted in tab-delimited format for further analysis. Records with missing demographic information or ambiguous ICD coding were excluded. Counts were cross-checked with CDC summary reports to ensure data consistency.

Sociodemographic variables included sex (male and female) and race, categorized according to the CDC classification as American Indian or Alaska Native, Asian or Pacific Islander, Black or African American, and White American. Geographic characteristics were also considered to explore mortality disparities. Place of death was classified based on the National Center for Health Statistics definitions into medical facility, home, hospice, nursing home/long-term care, and other locations [8]. In addition, urbanization level was determined using the 2013 Urban-Rural Classification Scheme for Counties, which categorizes counties as metropolitan or non-metropolitan [9]. This allowed the assessment of regional differences in mortality patterns related to population density and access to healthcare resources.

Mortality rates were calculated as crude and age-adjusted rates per 1,000,000 population. Age adjustment was performed using the 2000 U.S. standard population as the reference, which allows for standardized comparisons across years and subgroups [10]. Descriptive statistics and rate calculations were performed using Microsoft Excel 2021 (Microsoft Corp., Redmond, WA, US) and verified with the CDC WONDER interface. AAMR provide a more accurate representation of temporal trends by accounting for differences in population age structures over time. Absolute numbers and percentages were computed for all demographic and geographic variables to provide descriptive statistics of the study population.

Temporal trends in AAMR from 1999 to 2020 were analyzed using the Joinpoint Regression Program (Version 5.0.2; Statistical Methodology and Applications Branch, Surveillance Research Program, National Cancer Institute (NCI)) [11]. Joinpoint regression was applied to log-transformed AAMR to estimate the APC and 95% confidence intervals (CI). The best-fitting model, allowing a maximum of three joinpoints, was selected using the Monte Carlo permutation method (p<0.05), following standard NCI analytical procedures. Statistical significance was set at p<0.05.

All analyses were descriptive and exploratory, focusing on population-level mortality trends rather than hypothesis testing. Therefore, no additional inferential analyses were conducted beyond the estimation of APC values. The results were summarized using counts, percentages, and standardized mortality rates and visualized through graphical representations of trends by sex, race, and geographic category. As this analysis used a complete national dataset, selection bias was minimized. Missing data were limited to suppressed counts (<10) as per CDC privacy standards.

## Results

From 1999 to 2020, the CDC MCD database recorded 7829 deaths in the United States among individuals aged 25 years and older. Among these, deaths where thyroid disorder (ICD code: E00-E07) was listed as the underlying cause of death and cardiac arrest (ICD code: I46) was described as a multiple cause of death were included in the study (7829). The crude mortality rate for thyroid disorder with cardiac arrest as a contributing cause was 1.7 per 1,000,000 population. Deaths due to causes other than these criteria were excluded.

Demographic characteristics

As we have analysed the total deaths, women accounted for 5862 (74.90%), while men accounted for 1967 (25.10%). The mortality rate for thyroid disorder with cardiac arrest as a contributing cause was higher in female deaths, compared to male deaths, therefore indicating a potential demographic disparity.

Comparing racial distribution, the highest proportion of deaths occurred in the White individuals (n=6583, 84.10%), followed by the Black or African-American population (n=1022, 13.10%), individuals of Asian or Pacific Islander descent (n=184, 2.40%), and the American, Indian or Alaska native population (n=40, 0.50%).The mortality rate was highest among White individuals, highlighting racial disparities in mortality trends related to thyroid disorder and cardiac arrest.

Geographic characteristics

The maximum number of deaths occurred in the metropolitan areas (n=6418, 82%), whereas non-metropolitan areas accounted for 18% (n=1411) of deaths. When comparing the place of death, most deaths occurred in a medical facility (n=3282, 41.96%), followed by nursing home/long-term care (n=2280, 29.15%), decedent’s home (n=1903, 24.33%), other (n=303, 3.87%) and hospice facility (n=54, 0.69%).

Temporal trends

**Figure 1:**
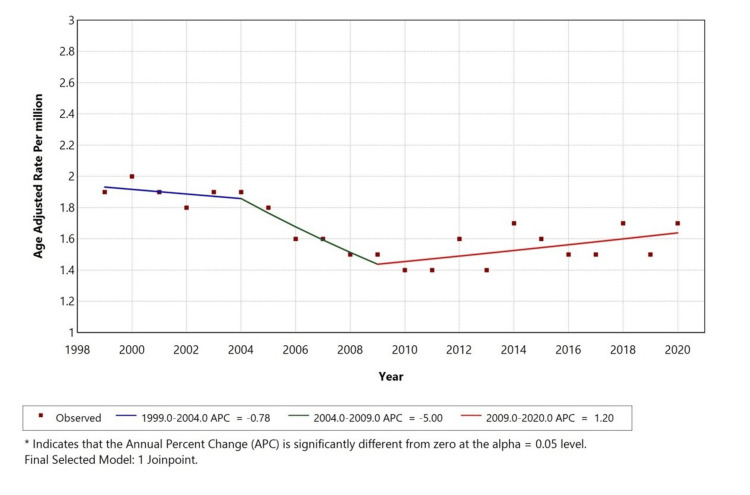
Overall age-adjusted mortality rates (AAMR) among adults aged 25+ in the United States (1999-2020) *Indicates that the annual percentage change (APC) is significantly different from zero at alpha=0.05 level. Figure created by the authors using the Joinpoint Regression Program (Version 5.0.2; Statistical Methodology and Applications Branch, Surveillance Research Program, National Cancer Institute (NCI)

From 1999 to 2020, the AAMR for thyroid disorder with cardiac arrest as a contributing cause initially showed a minor declining trend from 1999 to 2004 with an APC of -0.78%(p< 0.05). From 2004 to 2009, there was a further significant decline in AAMR with an APC of -5.00% (p<0.05.). In contrast, from 2009 to 2020, it began increasing slowly with an APC of +1.20 %( p<0.05). This shift indicates a notable change in mortality patterns over the past two decades (Figure 1).

**Figure 2:**
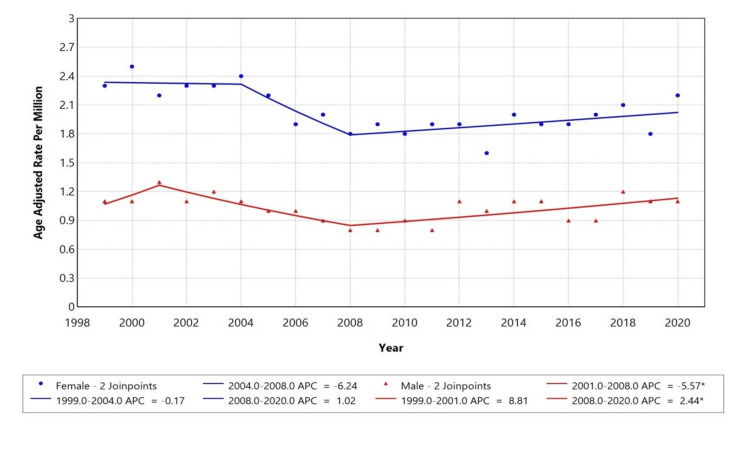
Trends in sex-stratified age-adjusted Mortality rates among adults aged 25+ in the United States (1999-2020) *Indicates that the annual percentage change (APC) is significantly different from zero at alpha=0.05 level. Figure created by the authors using the Joinpoint Regression Program (Version 5.0.2; Statistical Methodology and Applications Branch, Surveillance Research Program, National Cancer Institute (NCI)

When compared by gender, female deaths showed the fluctuating trend in AAMR. Between 1999 to 2004, the AAMR showed a decreasing trend (APC: -0.17%), followed by a significant decline from 2004 to 2008 (APC: -6.24%). However, after 2008, the AAMR began rising slowly (APC: +1.02%), indicating an upturn in mortality risk in the past decade. On the other hand, from 1999 to 2001, AAMR for male deaths showed an increase (APC: +8.81%), followed by a significant decline from 2001 to 2008 (APC: -5.57%). However, after 2008, it began rising (APC: +2.44%), indicating a fluctuation in mortality risk over the past decade (Figure 2).

**Figure 3:**
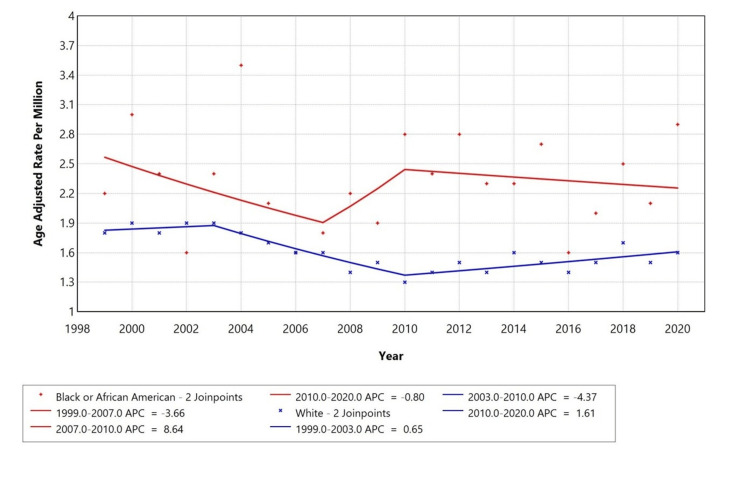
Trends in age-adjusted mortality rates stratified by race among adults aged 25+ years in the United States, 1999 to 2020. *Indicates that the annual percentage change (APC) is significantly different from zero at alpha=0.05 level. Temporal trends for American Indian/Alaska Native and Asian or Pacific Islander are not displayed due to data suppression for counts <10, limiting reliable trend analysis. Figure created by the authors using the Joinpoint Regression Program (Version 5.0.2; Statistical Methodology and Applications Branch, Surveillance Research Program, National Cancer Institute (NCI)

Racial disparities were observed in thyroid disorder with cardiac arrest as a contributing factor. Black or African-American individuals had a declining AAMR from 1999 to 2007 (APC: -3.66%); however, a further steep incline was observed from 2007 to 2010 (APC: +8.64%). This was again followed by a slow decline from 2010 to 2020 (APC: -0.80%). White individuals also showed an increase in AAMR from 1999 to 2003 (APC: +0.65%), followed by a significant decline from 2003 to 2010 (APC: -4.37%), an a slow increase from 2010 to 2020 (APC: +1.61%). Temporal trends for American Indian/Alaska native and Asian or Pacific Islander populations are not displayed due to data separation for counts <10, limiting reliable trend analysis (Figure 3).

## Discussion

This study was conducted retrospectively using data from the CDC-WONDER MCD database. Its primary objective was to examine mortality trends in thyroid gland disorders with cardiovascular disease and cardiac arrest as contributing factors in the United States from 1999 to 2020 among individuals aged 25 years and older. A total of 7,829 deaths were recorded. Mortality was higher among the female population and White individuals, with the greatest number of deaths occurring in metropolitan areas and medical facilities. AAMR for thyroid disorders with cardiovascular involvement and cardiac arrest showed a slight decline between 1999 and 2009, followed by a gradual increase from 2009 to 2020. Gender-specific analysis revealed that the female population experienced a decrease in AAMR from 1999 to 2008, after which rates began to rise. Similarly, White individuals showed a modest decline in AAMR from 2003 to 2010, but the overall trend was upward.

The present study found that thyroid gland disorders associated with cardiovascular disease and cardiac arrest had an overall AAMR of 1.7 per million. One study specifically examined mortality trends related to atrial fibrillation/atrial flutter (AF/AFL) in individuals with thyroid disorders between 1999 and 2020. Over this period, 7,187 deaths were reported, corresponding to an overall AAMR of 0.097 per 100,000 [12]. A meta-analysis reported that overt hyperthyroidism was linked to a 13% increase in overall mortality and a 21% increase in cardiovascular-related deaths [13]. Similarly, a more recent meta-analysis that included 37 studies and 113,393 patients with hyperthyroidism demonstrated that overt hyperthyroidism increases the risk of coronary heart disease (CHD), stroke, and cardiovascular mortality [14]. Evidence also indicates that even mild alterations in thyroid hormone levels can elevate cardiovascular mortality by 20-80% [15]. Findings from the NHANES conducted between 2007 and 2012 further support this association, showing that higher free thyroxine (FT4) levels were linked to increased all-cause mortality as well as cardiovascular mortality [16]. In the case of hypothyroidism, clinical research suggests that treatment with levothyroxine improves left ventricular contractility and function [17].

This study found that the metropolitan area group had the highest mortality compared to other groups. Women were observed to have a higher AAMR (74.90%) compared to men. A similar trend was noted in AF/AFL-related mortality among individuals with thyroid disorders, where women showed higher AF/AFL-related mortality than men [12]. In our study, the prevalence of mortality was highest among the White Americans (84.10%), while the lowest number of deaths was recorded among individuals from the American Indian or Alaska Native groups (0.50%). Zhang et al. reported similar findings, with the highest prevalence of thyroid disease observed in the non-Hispanic White population, in those aged ≥60 years, and more commonly in women [18]. According to a recent study, several population subgroups experienced disproportionately large increases in CVD mortality between 2019 and 2022, including younger and older adults, Black adults, and Asian or Pacific Islander adults [19]. These patterns are likely influenced by lifestyle risk factors, environmental exposures, and differences in healthcare access.

Geographic differences in mortality were evident, with urban areas showing the highest burden. In metropolitan regions, a total of 6,418 deaths (82%) were reported, compared with 846 deaths (10.80%) in micropolitan areas, and 565 deaths (7.20%) in non-core areas. Mortality also varied depending on the place of death. For instance, deaths occurring in medical facilities were the highest, accounting for 3,282 cases (41.96%), compared to deaths reported in other settings. This is opposed the observations seen in a 2025 U.S. study which demonstrated that CVD death rates were highest in high-poverty rural counties [20]. Another conflicting comparison includes a cohort study which found that, compared with urban patients, those living in rural and frontier areas were more likely to be diagnosed at later stages of thyroid cancer and had higher disease-specific mortality [21]. These findings suggest that demography plays a key role in shaping mortality patterns. This may be related to a combination of factors, such as greater population density, environmental exposures, healthcare system dynamics, and differences in lifestyle risk factors.

This retrospective study examined mortality trends in thyroid disorders with cardiovascular disease and cardiac arrest as the underlying cause, in the U.S. from 1999 to 2020, using the CDC WONDER database. Overall, AAMR showed a slight decline in the early years, followed by a gradual increase. Mortality was higher among women and White individuals, with women showing a decrease until 2008 before rising, and White individuals showing a modest decline from 2003 to 2010, but an overall upward trend. This study addresses an important gap in the literature, as comparable data from similar research is limited. These findings are therefore unique and provide a foundation for future investigations in this area. The observed mortality trends emphasize the need for further research into treatment-related disparities, patient outcomes, modifiable risk factors, and regional variations. Future studies should also examine mortality trends in thyroid disorders and their association with cardiovascular diseases to better inform prevention and management strategies.

The strengths of this study include the use of a large, nationally representative CDC WONDER dataset covering more than two decades, standardized ICD-10 coding, and validated Joinpoint regression analysis, which enhanced the reliability and generalizability of the findings. The study also provides sex-, race-, and geography-specific insights into mortality disparities.

However, certain limitations should be acknowledged. The ICD-10 codes E00-E07 encompass multiple thyroid disorders, preventing subtype-specific analyses. The descriptive design lacked a comparator group and did not adjust for potential confounders such as age, obesity, diabetes, or hypertension. Death-certificate data may include reporting variability or misclassification, and cardiac arrest may represent a terminal event rather than a causal mechanism. Although data verification was performed, differences in state-level reporting could influence trends. Finally, as a population-level analysis, these findings may not directly reflect individual risk (ecological limitation).

## Conclusions

This study examined mortality trends in thyroid disorders with cardiovascular disease and cardiac arrest in the U.S. from 1999 to 2020. Overall, mortality rates slightly declined in the early years but started rising after 2009. Women and White individuals had higher death rates, and most deaths occurred in metropolitan areas and medical facilities, highlighting clear demographic and geographic disparities. These findings point to the need for better public health strategies and clinical attention for high-risk groups. Healthcare providers should be aware of these trends when assessing risk and planning follow-up care. Future research should investigate the reasons behind these disparities, study treatment outcomes, and identify modifiable risk factors to improve prevention, early detection, and management of thyroid disorders and related cardiovascular complications.
